# LALF_32‐51_‐E7, a HPV‐16 therapeutic vaccine candidate, forms protein body‐like structures when expressed in *Nicotiana benthamiana* leaves

**DOI:** 10.1111/pbi.12802

**Published:** 2017-09-07

**Authors:** Romana J. R. Yanez, Renate Lamprecht, Milaid Granadillo, Isis Torrens, Elsa Arcalís, Eva Stöger, Edward P. Rybicki, Inga I. Hitzeroth

**Affiliations:** ^1^ Biopharming Research Unit Department of Molecular and Cell Biology University of Cape Town Cape Town South Africa; ^2^ Center for Genetic Engineering and Biotechnology Havana Cuba; ^3^ Department of Applied Genetics and Cell Biology University of Natural Resources and Life Sciences Vienna Austria; ^4^ Institute of Infectious Disease and Molecular Medicine University of Cape Town Cape Town South Africa

**Keywords:** protein bodies, chloroplasts, plant‐produced, membrane‐penetrating, HPV‐16, therapeutic vaccine, E7

## Abstract

High‐risk human papillomaviruses (HPVs) cause cervical cancer, and while there are good prophylactic vaccines on the market, these are ineffective against established infections, creating a clear need for therapeutic vaccines. The HPV E7 protein is one of the essential oncoproteins for the onset and maintenance of malignancy and is therefore an ideal therapeutic vaccine target. We fused the HPV‐16 E7 protein to the *Limulus polyphemus* antilipopolysaccharide factor (LALF_32‐51_), a small hydrophobic peptide that can penetrate cell membranes and that has immunomodulatory properties. LALF_32‐51_‐E7 was transiently expressed in *Nicotiana benthamiana*, and we previously determined that it accumulated better when targeted to chloroplasts compared to being localized in the cytoplasm. Subsequently, we aimed to prove whether LALF_32‐51_‐E7 was indeed associated with the chloroplasts by determining its subcellular localization. The LALF_32‐51_‐E7 gene was fused to one encoding enhanced GFP to generate a LG fusion protein, and localization was determined by confocal laser scanning microscopy and transmission electron microscopy (TEM). The fluorescence observed from chloroplast‐targeted LG was distinctively different from that of the cytoplasmic LG. Small spherical structures resembling protein bodies (PBs) were seen that clearly localized with the chloroplasts. Larger but less abundant PB‐like structures were also seen for the cytoplasmic LG. PB‐like structure formation was confirmed for both LG and LALF_32‐51_‐E7 by TEM. LALF_32‐51_‐E7 was indeed targeted to the chloroplasts by the chloroplast transit peptide used in this study, and it formed aggregated PB‐like structures. This study could open a new avenue for the use of LALF_32‐51_ as a PB‐inducing peptide.

## Introduction

Recombinant proteins have a wide range of applications in the industrial, biomedical and biological research fields. There is a high demand for recombinant proteins, which in turn requires efficient and scalable production systems. Current methods of production of these proteins are mostly based on the use of mammalian, insect or microbial cell cultures, which necessarily rely on bioreactors and on expensive growth media. Plants provide an alternative platform for the expression of recombinant proteins that is potentially more cost‐effective than conventional methods and that is also highly scalable (Fischer, 2013; Pogue and Holzberg, 2012; Rybicki, 2009; Sainsbury *et al*., 2007; Yusibov *et al*., 2006).

One of the factors that can significantly increase yields in plant expression systems is the specific subcellular localization of the recombinant protein. Different compartments can be used, such as chloroplasts, the endoplasmic reticulum (ER), storage vacuoles, the apoplast and peroxisomes, depending on the requirements of the target protein (Benchabane *et al*., 2008; Fischer *et al*., 2004; Karg and Kallio, 2009; Maclean *et al*., 2007; Meyers *et al*., 2008; Streatfield *et al*., 2003). Of these, chloroplast localization is one of the most used and most successful options for increasing the accumulation of specific proteins (Daniell *et al*., 2009; Hofbauer *et al*., 2014; Lakshmi *et al*., 2013; Zahin *et al*., 2016).

For subcellular localization after nuclear transcription, whether this is in stable nuclear transformants or transiently transformed plant cells, transit peptides are genetically fused to the protein of interest. The proteins are translated in the cytoplasm of the plant cells and subsequently transported to the target organelle. The generally higher yields obtained by transient expression, combined with a further increase in protein accumulation and protein stability away from the cytoplasm, make this an attractive prospect for molecular farming (Fischer *et al*., 2004; Rybicki, 2010). In particular, our group has found the use of the *rbcS1* gene‐derived chloroplast transit peptide (cTP) from the RuBisCO small subunit of *Solanum tuberosum* to be highly effective for improving the accumulation of a number of recombinant proteins (Maclean *et al*., 2007; Meyers *et al*., 2008).

High‐risk human papillomaviruses (HPVs) cause 99.7% of cervical cancer cases (Parkin and Bray, 2006; de Villiers, 2013; Zur Hausen, 2002). There are approximately 530 000 new cases of cervical cancer and 270 000 deaths per year (World Health Organization 2016). HPV‐16 and ‐18 are the most prevalent types worldwide, causing more than 70% of cervical cancer cases. Besides cervical cancer, high‐risk HPVs also cause vaginal, vulvar, penile, anal and oropharyngeal cancers (Parkin and Bray, 2006; Zur Hausen, 2002, 2009).

Currently, there are three commercially available prophylactic vaccines for HPVs, all of which consist of the HPV major coat protein L1 assembled into virus‐like particles specific for target HPV types. The vaccines are the bivalent (HPV‐16 + 18) Cervarix^®^ (GlaxoSmithKline Inc., Brentford, London, UK), the quadrivalent (HPVs 6, 11, 16 and 18) Gardasil^®^ (Merck & Co., Kenilworth, New Jersey, US) and the new nonavalent Gardasil^®^ 9 (Merck 2014). However, these vaccines are not effective in eliminating pre‐existing infections (Hildesheim *et al*., 2007, 2016; Hung *et al*., 2008). Therefore, the large number of individuals already infected with HPVs, and those who have developed malignancies, do not benefit from the current vaccines. In addition, these vaccines are expensive and unless sponsored by the state or other organizations, they are not accessible to low‐income populations. New types of vaccines are therefore needed that can eliminate established infections, and that are also more accessible to poorer communities (Giorgi *et al*., 2010; Hildesheim *et al*., 2007).

The HPV E7 protein is one of the two viral oncoproteins that are essential for the onset and maintenance of malignancy, and is therefore an ideal therapeutic vaccine target. Granadillo *et al*. (2011) developed a HPV‐16 therapeutic vaccine candidate consisting of the HPV‐16 E7 oncoprotein fused to a peptide derived from the *Limulus polyphemus* antilipopolysaccharide factor (LALF_32‐51_). This is a small and hydrophobic peptide that can penetrate cell membranes and that has immunomodulatory properties (Granadillo *et al*., 2011, 2015). The fusion to HPV‐16 E7 improved both the immunogenicity and antigen presentation of E7 in animal experiments, and LALF_32‐51_‐E7 showed promising results for the treatment of already established HPV‐16 infections and tumours (Granadillo *et al*., 2011, 2015).

Accordingly, we investigated the expression of LALF_32‐51_‐E7 in *Nicotiana benthamiana* leaves as an alternative to bacterial expression systems, for providing a more accessible HPV therapeutic vaccine candidate. We targeted it to the chloroplasts to determine whether that would enhance accumulation, as we previously detected only very low expression levels when the protein was targeted to the cytoplasm. Indeed, LALF_32‐51_‐E7 accumulated to higher levels when targeted to the chloroplasts of *N. benthamiana* leaves (up to 0.56% of total soluble proteins, TSP) than when not targeted to any cell compartment (up to 0.017% TSP) (Yanez *et al.,* 2017).

In order to prove that this protein was indeed being targeted to the plant chloroplasts, it was fused to EGFP and its subcellular localization was determined by fluorescence confocal laser scanning microscopy (CLSM). As LALF_32‐51_ is a cell membrane‐penetrating peptide (Granadillo *et al*., 2011), it was also desirable to determine whether LALF_32‐51_‐E7 interacted with the plant cell interior membranes.

## Results

### Optimization of LG expression in *N. benthamiana* leaves

**Figure 1:**
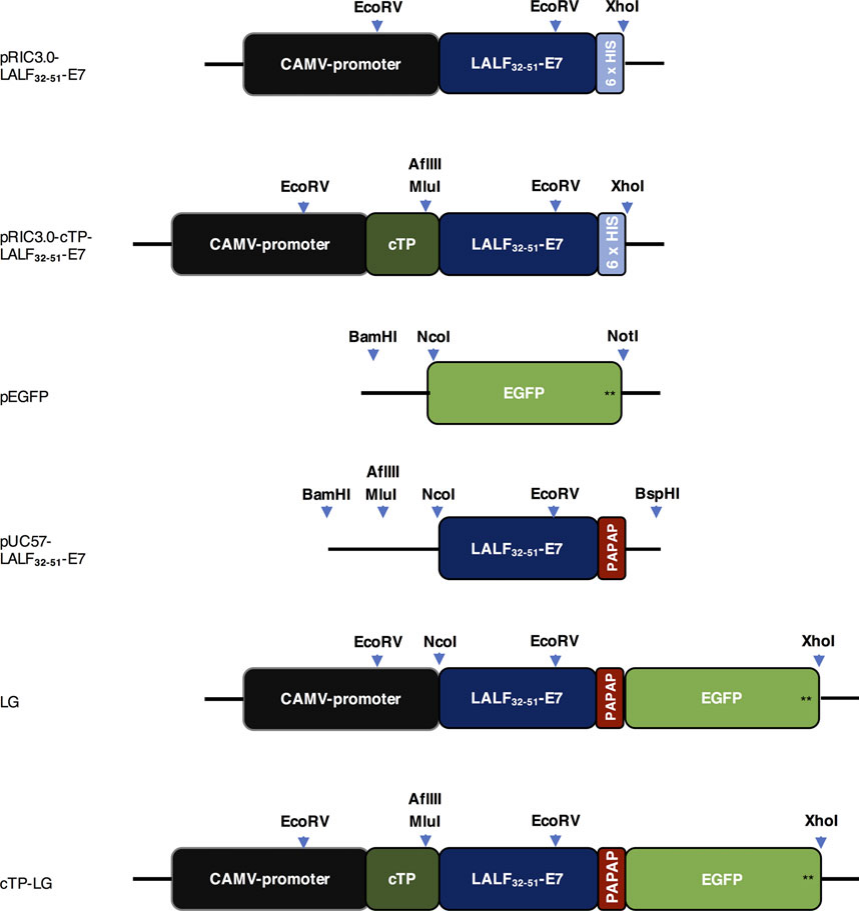
Representation of the relevant constructs used in this work. pRIC3.0‐LALF_32‐51_‐E7 and pRIC3.0‐cTP‐LALF_32‐51_‐E7 are the initial constructs used to express LALF_32‐51_‐E7 in *N. benthamiana* leaves. pEGFP contains the target *EGFP* sequence and a stop codon (black stars). pUC57‐LALF_32‐51_‐E7 contains the plant codon‐optimized *LALF‐E7* sequence, appropriate restriction sites for subcloning (shown by the light‐blue arrows), a rigid linker (*PAPAP*) and no His‐tag or stop codons. The final constructs pRIC3.0‐LALF_32‐51_‐E7‐EGFP (LG) and pRIC‐cTP‐LALF_32‐51_‐E7‐EGFP (cTP‐LG) were generated in a stepwise manner. Also shown are the cauliflower mosaic virus (CAMV) 35 S constitutive promoter and the chloroplast transit peptide (*cTP*). Not drawn to scale.

The LALF_32‐51_‐E7 gene was genetically fused to one encoding the enhanced green fluorescent protein (EGFP), creating the LG gene construct. This was cloned into the replicative vectors pRIC3.0 and pRIC3.0‐cTP, creating the constructs LG and cTP‐LG, respectively (Figure 1). Final constructs were then electroporated into competent *Agrobacterium* cells.

**Figure 2:**
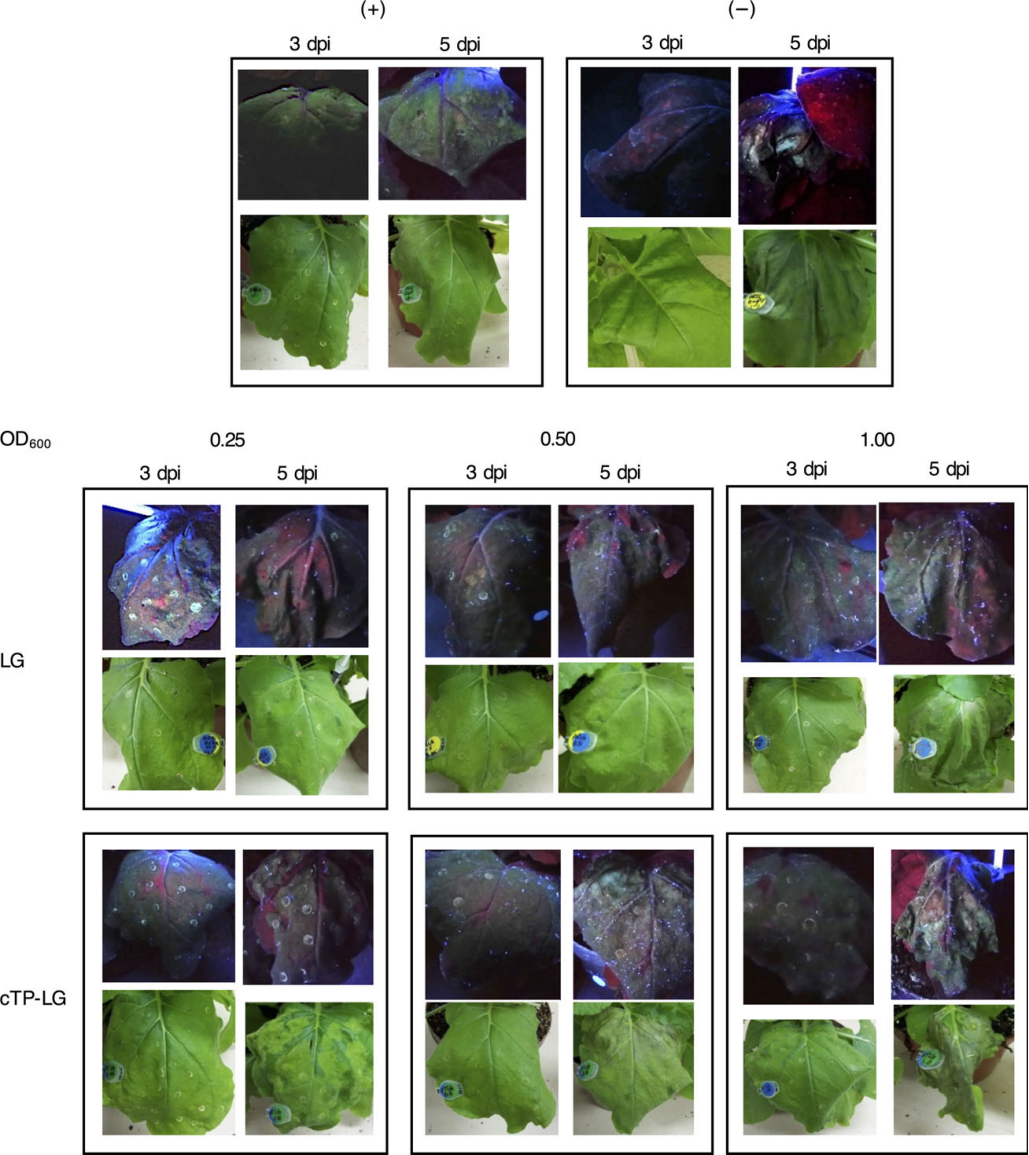
Optimization of LG expression in syringe‐infiltrated *N. benthamiana* leaves. Plants infiltrated with pRIC3.0‐EGFP and pRIC3.0 empty vector were used as positive (+) and negative (−) controls, respectively. Experimental plants were infiltrated with LG and cTP‐LG at different OD_600_. Shown here is a representative picture of each vector/OD_600_ combination under UV‐light (top) and white light (bottom).

To determine the best conditions for the expression of LG in *N. benthamiana* leaves, *Agrobacterium* suspensions of different optical densities (OD_600_) were used to syringe‐infiltrate leaves, and LG expression was monitored for 5 days postinfiltration (dpi) by UV‐light visualization. Leaves infiltrated with the positive control pRIC3.0‐EGFP fluoresced green throughout the experiment, as expected (Figure 2). Uninfiltrated leaves and leaves infiltrated with an empty version of the pRIC3.0 vector fluoresced red, except in areas of leaf damage such as at the points of infiltration where a white‐blue fluorescence was seen 5 dpi. Leaves infiltrated with LG and cTP‐LG fluoresced green on 3 and 5 dpi regardless of the OD_600_ used. However, higher OD_600_ and longer incubation periods were associated with more damage symptoms and lower green fluorescence.

For fluorescence confocal laser scanning microscopy (CLSM), healthy undamaged leaf tissue was desirable. Therefore, OD_600_ of 0.25–0.5, and 3 dpi were considered optimal conditions for LG expression from both vectors.

### Confirmation of LG expression by immunoblotting

**Figure 3:**
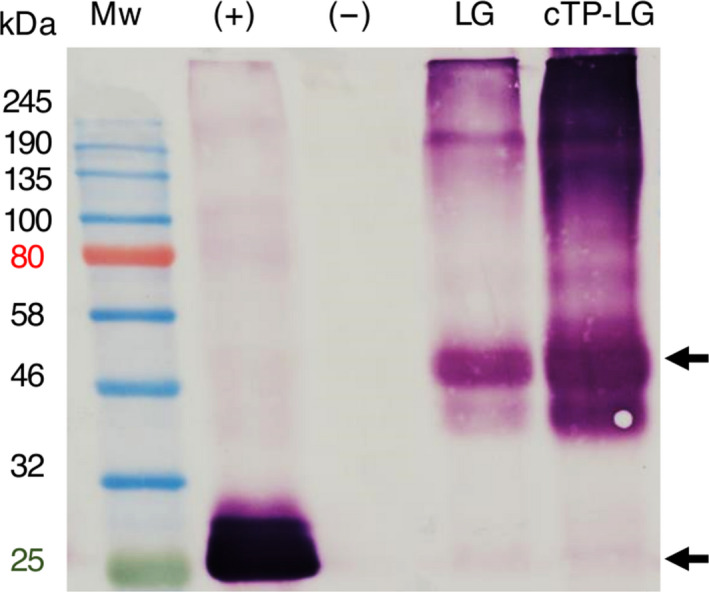
Immunoblot of LG and cTP‐LG leaf crude extracts. Crude extracts were prepared of vacuum‐infiltrated *N. benthamiana* leaves. (+), positive control, pRIC3.0‐EGFP. (−), negative control, pRIC3.0 empty vector. Mw, protein molecular weight marker. Sizes are shown on the left hand side in kilodaltons (kDa). Arrows point at the position of LG ≈ 50 kDa and EGFP ≈ 25 kDa. Primary and secondary antibodies: 1 : 5000 monoclonal anti‐GFP and anti‐mouse, respectively.

The expression of LG was confirmed by anti‐GFP immunoblots of LG and cTP‐LG crude extracts. The LG‐ and cTP‐LG‐containing extracts showed the same banding patterns, with both displaying high molecular weight aggregates that were not disrupted by boiling with sodium dodecyl sulphate (SDS) and beta‐mercaptoethanol and that were larger than 190 kDa. Furthermore, the most distinct band for both samples appeared at the same molecular weight, of approximately 50 kDa (Figure 3).

### Subcellular localization of LG by fluorescence confocal laser scanning microscopy

Once the optimal conditions were determined for the expression of LG, leaves were vacuum‐infiltrated with *Agrobacterium tumefaciens* grown to the optimal OD_600_ and were analysed by confocal laser scanning microscopy at 3 dpi. Young plants were used as their leaves are thinner than those of older plants: this allowed for appropriate leaf sectioning and better visualization.

**Figure 4:**
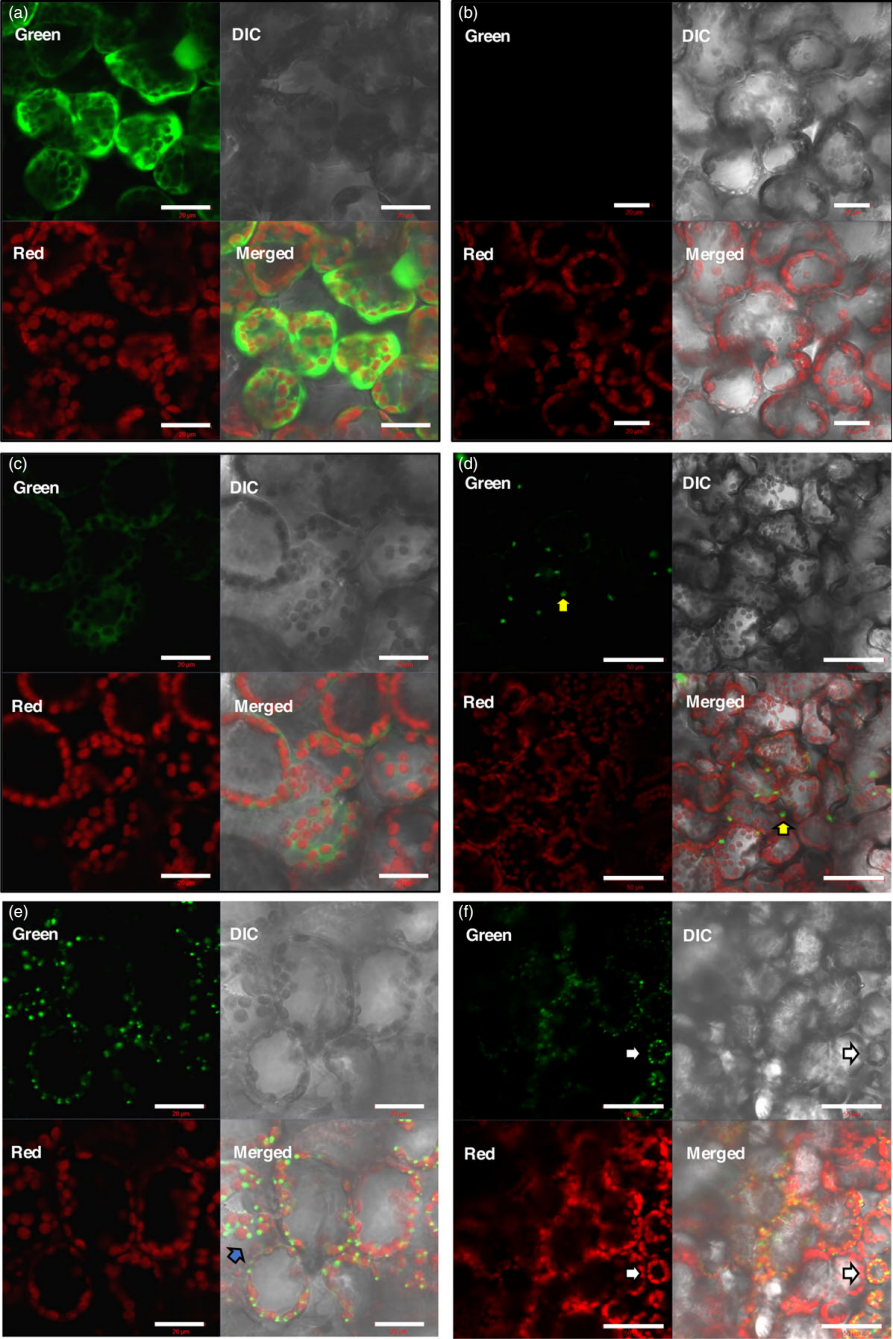
Fluorescence CLSM images of the subcellular localization of LG. *N. benthamiana* leaves were vacuum‐infiltrated with the relevant constructs. On 3 dpi, live leaf sections were prepared for visualization. (a) Positive control, pRIC3.0‐EGFP. (b) Negative control, pRIC3.0 empty vector. (c–d) Cytoplasmic LG. (e–f) cTP‐LG. Green channel, EGFP fluorescence. Red channel, chlorophyll autofluorescence. DIC, differential interference contrast. Merged, an overlap of the green, red and DIC channel images. For (a)–(c) and (e): scale bar = 20 μm, magnification = 40×. For (d) and (f): scale bar = 50 μm, magnification = 20×. Shown here are representative of several images taken over two independent sets of experiments.

In cells expressing the positive control, green fluorescence could be seen evenly distributed in the cytoplasm. No green fluorescence was seen inside the chloroplasts as these appeared as black or empty spheres in the green channel, and no yellow colour was detected when the green and red channels were merged. This was typical of a cytoplasm‐localized protein (Figure 4a). No green fluorescence was observed in samples of the negative controls, pRIC3.0 empty vector (Figure 4b) and uninfiltrated leaves (not shown), as expected, and only chloroplasts were seen by red autofluorescence.

The fluorescence pattern in the cytoplasmic LG samples resembled that of the positive control; however, it was less intense (Figure 4c), as previously observed in the optimization experiments. Besides the evenly spread green fluorescence within the plant cells, large bright globular structures could also be seen that resembled protein bodies (PBs; Figure 4d, yellow arrows). These globular structures were not as common as the ones seen for the cTP‐LG, but they were observed in two rounds of independent experiments.

Interestingly, cells expressing the cTP‐LG presented a very different fluorescence pattern. The cTP‐LG was seen as numerous bright dots also resembling PBs, but which were smaller and more abundant than those seen in some cytoplasmic LG samples (Figure 4e–f). The distribution of these PB‐like structures followed the distribution of the chloroplasts, and they appeared yellow when seen at a lower magnification (Figure 4f, white arrows). When inspected at a higher magnification, the cTP‐LG PB‐like structures appeared to be mostly at the periphery of the chloroplasts (Figure 4e, blue arrow). However, no cTP‐LG was seen separated from chloroplasts, even though chloroplasts that did not have cTP‐LG PB‐like structures associated with them could be seen.

### Colocalization profiles of LG and cTP‐LG with chloroplasts

**Figure 5:**
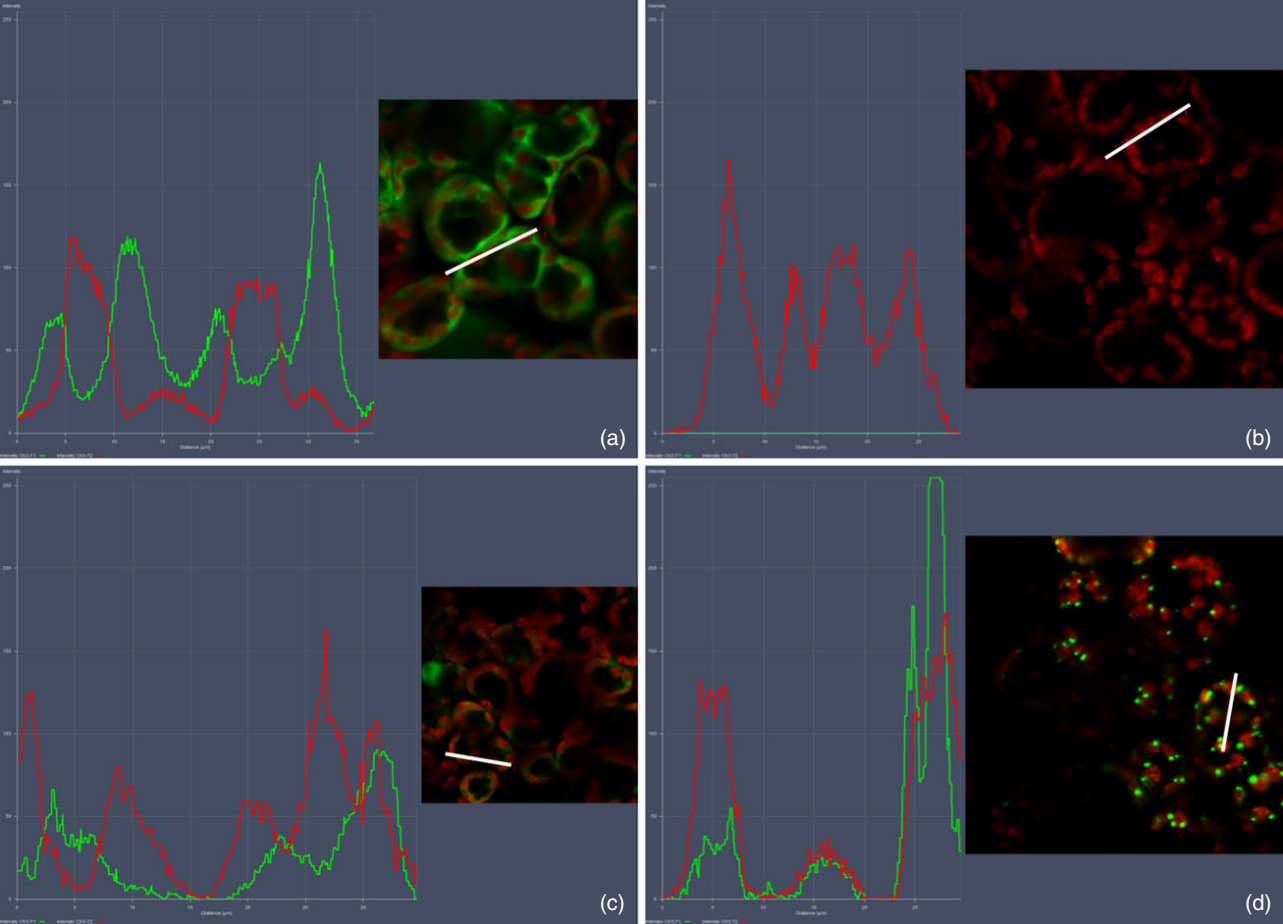
Colocalization profiles of LG and chloroplasts. Colocalization profiles were generated using the colocalization feature of the Zeiss Zen software. (a) Positive control, pRIC3.0‐EGFP. (b) Negative control, pRIC3.0 empty vector. (c) Cytoplasmic LG. (d) cTP‐LG. Green and red curves correspond to EGFP fluorescence and the chlorophyll autofluorescence, respectively. White bars represent the area used to generate the colocalization profiles. Magnification = 40×. Shown here are representatives of several images taken over two independent sets of experiments.

Fluorescence microscopy images were analysed using the colocalization settings on the Zeiss microscope Zen software (Figure 5). In the positive control, overlapping of the red and green fluorescence curves was not common, as expected, indicating that the EGFP was not localized inside the chloroplast (Figure 5a). The negative control gave only a red fluorescence curve (Figure 5b). The curve pattern for the cytoplasmic LG was similar to that of the positive control, except that the green fluorescence intensity was lower (Figure 5c). The green and red fluorescence curves for the cTP‐LG, however, followed the same pattern—as one increased, so did the other. This indicated that the cTP‐LG was associated with the chloroplasts (Figure 5d). Here, the green fluorescence curve was at times very high, suggesting that the PB‐like structures seen for the cTP‐LG contained very concentrated LG.

### Confirmation of protein body‐like structure formation by transmission electron microscopy

**Figure 6:**
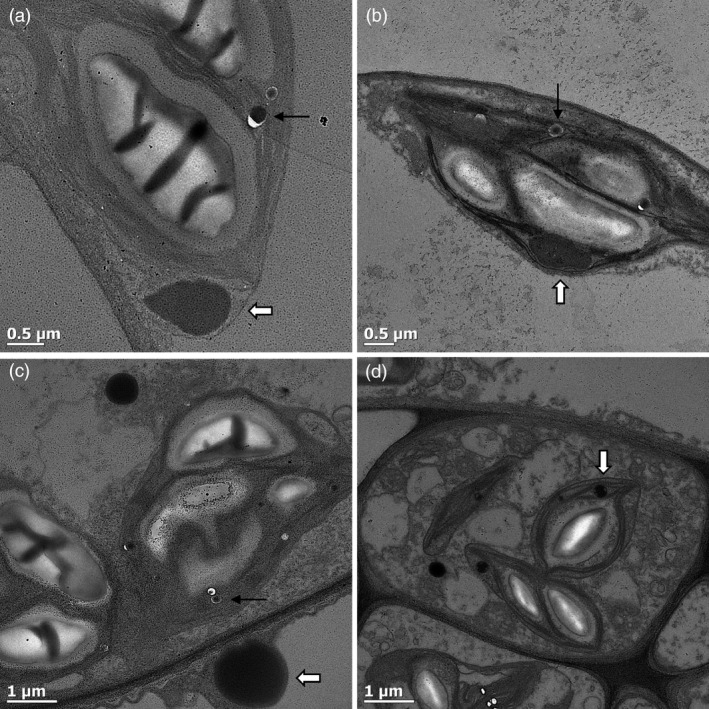
Confirmation of LALF_32‐51_‐E7 protein body formation. Agro‐infiltrated leaves were fixed and positively stained for visualization under a TEM. (a) pRIC3.0‐LALF_32‐51_‐E7. (b) pRIC3.0‐cTP‐LALF_32‐51_‐E7. (c) LG. (d) cTP‐LG. White arrows point to protein bodies. Black arrows point to oil droplets. Shown here are representatives of several images captured.

To confirm whether LALF_32‐51_‐E7 and LG formed PB‐like structures, agro‐infiltrated leaves were fixed and positively stained for visualization by transmission electron microscopy (TEM). Large protein aggregates were observed for the cytoplasmic LALF_32‐51_‐E7 and cTP‐LALF_32‐51_‐E7. These were electron‐dense, irregular or spherical in shape and larger than 1 μm (Figure 6a–b, white arrows). Similar structures were observed for LG and cTP‐LG: these were larger than 1 μm for the cytoplasmic LG and smaller for the cTP‐LG (Figure 6c–d). These structures were electron‐dense and contained electron‐lucent patches. These results confirmed that LALF_32‐51_‐E7 forms PB‐like structures. It could be seen that the cTP‐LALF_32‐51_‐E7 and cTP‐LG aggregates were located between the thylakoid stacks within the chloroplasts; however, it was not possible to determine whether the structures observed were membrane‐bound.

Spherical structures that were highly electron‐dense and smaller than the LALF_32‐51_‐E7 PB‐like structures were observed in all samples (Figure 6, black arrows). These were identified as oil droplets and were also present in negative control samples (not shown).

### Predicted targeting peptides encoded within the LALF_32‐51_‐E7 and LG amino acid sequences

To confirm whether the observations described above were not a result of cryptic targeting peptides present within the LALF_32‐51_‐E7 and LG amino acid sequences, these were analysed using the TargetP and ChloroP prediction programmes (http://www.cbs.dtu.dk/services/TargetP/; http://www.cbs.dtu.dk/services/ChloroP/). No targeting sequences were identified when the LALF_32‐51_‐E7 and LG amino acid sequences were submitted. However, when the cTP‐LALF_32‐51_‐E7 and cTP‐LG amino acid sequences were submitted, the presence of a cTP was indicated and a chloroplast localization was predicted with RC‐values of 1 and 2, respectively. This indicated that the prediction was carried out with a high degree of confidence.

## Discussion

The production of accessible HPV vaccines is of particular relevance for the developing world, where HPV infections and related malignancies are most prevalent (Giorgi *et al*., 2010; Stanley, 2006). For this reason, we expressed LALF_32‐51_‐E7, a HPV‐16 therapeutic vaccine candidate, using plants as an alternative expression platform.

We found that the accumulation levels of LALF_32‐51_‐E7 in *N. benthamiana* leaves were enhanced by 27‐fold when targeted to the chloroplasts, compared to protein not targeted to a cell compartment. In order to prove that LALF_32‐51_‐E7 was in fact being targeted to the chloroplasts, we fused it to EGFP. As LALF_32‐51_ is a cell membrane‐penetrating peptide, it could be possible that it would be associated with the plants’ cell membranes or that it could in fact exit the plant cells. Therefore, determining its subcellular localization seemed to be imperative.

From the images taken during fluorescence CLSM, it could be seen that LG expressed from pRIC3.0 behaved similarly to the positive control, by localizing like a typical cytoplasmic protein. It could also be seen that it formed large PB‐like aggregates; however, these were less common and more heterogeneous than those seen in the cTP‐LG samples. It has been shown that LALF_32‐51_‐E7 forms inclusion bodies in *Escherichia coli* cells and that it forms large molecular weight aggregates of varying sizes, even after its purification (Granadillo *et al*., 2013), so this phenomenon was not surprising. This sample also showed a less intense fluorescence than the cTP‐LG, agreeing with previous accumulation levels obtained for the untagged cytoplasmic LALF_32‐51_‐E7.

The fluorescence pattern seen for the cTP‐LG was distinctively different from that of the cytoplasmic LG. The small spherical structures seen for the cTP‐LG clearly colocalized with chlorophyll, confirming the association with the chloroplasts. These spherical structures were also comparable in shape and size to the PBs formed by the cTP‐γ‐Zein‐DsRed fusion protein seen by Hofbauer *et al*. (2014), which were induced by adding the N‐terminal portion of mature 27 kDa γ‐zein to a reporter protein. These authors reported ectopic PBs found in the stroma and in the intermembrane space of chloroplasts, demonstrating that PB formation and budding do not require ER‐specific factors and can happen in different subcellular localizations. The present study suggests that LALF_32‐51_ can also induce PB formation. Although LALF_32‐51_ is much shorter than the N‐terminal portion of mature 27 kDa γ‐zein, both sequences share an amphipathic character and contain cysteine residues, possibly initiating aggregates by hydrophobic interactions.

Bross *et al*. (2017) observed similar globular structures when they investigated localization of arogenate dehydratases (ADT) fused to cyan fluorescent protein in *N. benthamiana*. They postulate that the ADTs were targeted to stromules, which are stroma‐filled protrusions of the outer and inner membrane from chloroplasts (Bross *et al*., 2017). The similarity of these globular structures to the ones observed in our study suggests that the cTP‐LG might be targeted to stromules.

Scatter plots of the colocalization of EGFP or LG and chloroplasts were generated. These showed colocalization for all samples except for the negative control, which was not surprising as the EGFP and LG were within the cells’ cytoplasm, surrounding the chloroplasts and other organelles. Fluorescence profiles proved to be more useful: these further illustrated that the cTP‐LG was associated with chloroplasts, while no association was seen for the cytoplasmic LG.

Taken together, the fluorescence microscopy results showed that the cTP signal indeed targeted LALF_32‐51_‐E7 to the chloroplasts. Furthermore, immunoblots of LALF_32‐51_‐E7 and cTP‐LALF_32‐51_‐E7 (not shown) as well as LG and cTP‐LG show these proteins at the same molecular weights of approximately 22 and 50 kDa, respectively. This further confirms that the cTP‐LALF_32‐51_‐E7 and cTP‐LG entered the chloroplasts, as this is an indication that the cTP signal was cleaved during the membrane translocation into the chloroplast stroma (Li and Chiu, 2010).

Chlorophyll pigments are located within the thylakoid membranes in the stromal compartment of chloroplasts. At the resolution at which the fluorescent CLSM images were taken here, it was not possible to differentiate between the chloroplast internal compartments. However, our TEM observations suggest that the cTP‐LG and cTP‐LALF_32‐51_‐E7 PB‐like structures are located in the stromal compartment, mostly at a peripheral position within the plastid.

These findings suggest that the LALF_32‐51_ peptide is a potential PB‐inducer. If this is the case, LALF_32‐51_ could have significant promise as a fusion partner which enhances the accumulation of other recombinant proteins in plant expression systems, similar to the Zera^®^ peptide, elastin‐like polypeptides (ELP) and hydrophobin (Conley *et al*., 2011; Saberianfar *et al*., 2016). Its small size is also an advantage. Furthermore, LALF_32‐51_‐fusion proteins could also be targeted to the ER in order to possibly generate larger and more numerous PBs. This would in turn further enhance the accumulation levels and stability of recombinant proteins as well as simplifying their purification processes (Conley *et al*., 2011; Hofbauer *et al*., 2014; Torrent *et al*., 2009; Whitehead *et al*., 2014).

In summary, we have shown how LALF_32‐51_‐E7 and LALF‐E7‐GFP polypeptides form PB‐like structures when targeted to chloroplasts, which are more concentrated and abundant than for the cytoplasmic versions. These results could explain why LALF_32‐51_‐E7 accumulated to much higher levels when targeted to the chloroplasts than when localized in the cytoplasm in our vaccine production feasibility study (Yanez *et al.,* 2017).

This study was a proof of concept that confirms the importance of subcellular localization, and which further highlights that chloroplasts are a useful compartment to which to target recombinant proteins. This study also opened new avenues for the use of the LALF_32‐51_ peptide as a potential PB‐inducer.

## Experimental procedures

### Bacterial strains and growth conditions

#### Escherichia coli

All constructs were maintained in DH5‐α chemically competent *E. coli* cells (*E. cloni™,* Lucigen). Cells were grown in Luria‐Bertani (LB) medium [1.0% tryptone, 0.5% yeast extract, 1.0% NaCl, pH 7.0]. Antibiotic selection was made using 100 μg/mL ampicillin.

#### Agrobacterium tumefaciens

The strain GV3101 containing the helper plasmid pMP90RK was used. Cells were grown in LBB‐enriched medium [0.25% tryptone, 1.25% yeast extract, 0.50% NaCl, 10 mm 2‐(N‐Morpholino) ethanesulphonic acid (MES), pH 5.6], with agitation at 120 r.p.m., at 27 °C for 2–3 days. Antibiotic selection was made using 50 μg/mL carbenicillin, 50 μg/mL rifampicin and 30 μg/mL kanamycin.

### Construct generation

The sequence encoding LALF_32‐51_‐E7 was kindly made available by the Center for Genetic Engineering and Biotechnology, Havana. The E7 coding sequence was previously modified to contain a base substitution (T/G) in the codon encoding for the first cysteine to abolish the carcinogenic effect of the protein (Granadillo *et al*., 2011). The LALF_32‐51_ sequence encodes a small linear peptide containing residues 32–51 from the original LALF protein (Vallespi *et al*., 2004). To fuse LALF_32‐51_‐E7 to EGFP, the DNA sequence encoding LALF_32‐51_‐E7 was modified to remove the hexa‐histidine tag and the stop codon, to add appropriate restriction enzyme (RE) sites and to insert a linker between the two peptides, as shown in Figure 1. The modified *LALF*_*32‐51*_ *‐E7* was plant codon‐optimized and synthesized by GenScript (Nanjing, China). LALF_32‐51_‐E7 was genetically fused to EGFP, and the resulting LG fragment was subcloned into the expression vectors pRIC3.0 and pRIC3.0‐cTP using the NcoI/AflIII and the MluI/XhoI restriction sites, respectively.

Electro‐competent *A. tumefaciens* cultures were prepared as described by Wen‐jun and Forde (1989) and electroporated as described by Maclean *et al*. (2007).

### Agroinfiltration of *N. benthamiana* leaves

Recombinant *A. tumefaciens* cultures were prepared for small‐scale agroinfiltration as described by Maclean *et al*. (2007). For small‐scale expression studies, *A. tumefaciens* cultures were diluted in infiltration medium to final OD_600_s of 0.25, 0.50 and 1.00 to determine the optimal OD_600_ for the expression of LG. Two plants were used per construct/OD_600_ combination. A time trial was carried out to determine the expression profile of pR‐LG and pT‐LG, as well as to monitor leaf symptoms. This was done by visualizing infiltrated leaves under white light and ultraviolet light (Spectroline Long Life™ Filter, set at 365 nm) on 3 and 5 dpi, and harvesting plant tissue on the same time points. The construct pRIC3.0‐EGFP was used as a positive control at an OD_600_ of 0.25 as determined by Regnard *et al*. (2010). As negative control, the pRIC3.0 empty vector was used at an OD_600_ of 0.5. One plant was used per control. For the constructs, pRIC3.0‐LALF_32‐51_‐E7 and pRIC‐cTP‐LALF_32‐51_‐E7, *A. tumefaciens* suspensions at OD_600_ of 1.0 were used and leaves were harvested on 3 dpi.

For fluorescence CLSM, young *N. benthamiana* plants, 3–4 weeks old, were vacuum‐infiltrated with *A. tumefaciens* cultures at an OD_600_ of 0.25. *Agrobacterium* cultures were grown as described by Maclean *et al*. (2007) in LBB‐enriched medium, induced overnight with 20 μm acetosyringone and diluted to the desired final OD_600_ in resuspension solution [5 mm MES, 20 mm MgCl_2_, 0.2 mm acetosyringone].

### Detection of LG by immunoblotting

Protein extraction was carried out by homogenizing leaf material in 2 v/w extraction buffer [8 m urea in 1 mm carbonate‐bicarbonate buffer, pH 10.6]. For immunoblotting, the primary and secondary antibodies used were mouse monoclonal anti‐GFP (Sigma‐Aldrich, St Louis, MO, US) and goat anti‐Mouse IgG whole molecules conjugated to alkaline phosphatase (AP; Sigma‐Aldrich), respectively, at a dilution of 1 : 5000.

### Fluorescence confocal laser scanning microscopy (CLSM)

Vacuum‐infiltrated leaves were manually sectioned with a razor blade into very fine strips and mounted in distilled water on a glass slide. A cover slip was placed on top of the sections for visualization. Thin leaf sections were imaged using a Zeiss LSM 510 Meta NLO multiphoton confocal microscope and the ZEN 2009 software (Zeiss). The lenses used included a 20× air lens and a 40× water immersion lens (numerical aperture of 1.1). The EGFP was excited by an argon laser at 488 nm, and the emission was detected at 500–550 nm. The chloroplasts were indirectly detected by exciting the chlorophyll by a DPSs laser at 561 nm, and the emission was detected at 650–710 nm. Imaging parameters were fixed for all data acquisition for both rounds of microscopy done.

For colocalization studies, images obtained during sample visualization were analysed using the colocalization set‐up on the ZEN software. To accurately set the cross‐hairs, single label control samples must be prepared. For the red label sample (chloroplasts), the negative control was used. Due to the nature of the samples, we did not have a green single label. Therefore, the positive control was used. These controls were imaged with the same microscope settings as the experimental sample. All imaging settings, including the crosshair positions, were kept fixed throughout the colocalization analysis.

### Transmission electron microscopy

Agro‐infiltrated leaf tissue was cut into 1 mm by 3‐mm sections and fixed with a 2.5% glutaraldehyde solution overnight (O/N) at 4 °C. Leaf sections were washed in 0.1 m phosphate buffer pH 7.2. Leaf sections were further fixed by a 1% osmium tetroxide in phosphate buffer, for 1 h at room temperature (RT), followed by dehydration by sequential immersion in ethanol–acetone solutions of higher content. Finally, the samples were included in Spurr's resin by sequential immersion in resin–acetone mixtures of increasing percentages of resin. Ultra‐thin sections were positively stained with uranyl acetate and lead citrate and viewed using a JEOL 200CX transmission electron microscope.

### Prediction of targeting peptides encoded within LALF_32‐51_‐E7 and LG

The TargetP and ChloroP server software from the Technical University of Denmark (http://www.cbs.dtu.dk/services/TargetP/; http://www.cbs.dtu.dk/services/ChloroP/) were used to analyse the amino acid sequences of LALF_32‐51_‐E7 and LG, with and without the cTP amino acid sequence.

## Conflict of interest

The authors declare no conflict of interest.
